# Cranberry fruit epicuticular wax benefits and identification of a wax-associated molecular marker

**DOI:** 10.1186/s12870-023-04207-w

**Published:** 2023-04-05

**Authors:** Lindsay Erndwein, Joseph Kawash, Sara Knowles, Nicholi Vorsa, James Polashock

**Affiliations:** 1 ORISE Postdoctoral Research Associate, Chatsworth, NJ 08019 USA; 2 Genetic Improvement of Fruit and Vegetables Laboratory, Agricultural Research Service, USDA-ARS, Chatsworth, NJ 08019 USA; 3 P.E. Marucci Center for Blueberry and Cranberry Research and Extension, Rutgers University, Chatsworth, NJ 08019 USA

**Keywords:** Fruit wax, Cuticle, QTL, *Vaccinium macrocarpon*, Genotyping

## Abstract

**Background:**

As the global climate changes, periods of abiotic stress throughout the North American cranberry growing regions will become more common. One consequence of high temperature extremes and drought conditions is sunscald. Scalding damages the developing berry and reduces yields through fruit tissue damage and/or secondary pathogen infection. Irrigation runs to cool the fruit is the primary approach to controlling sunscald. However, it is water intensive and can increase fungal-incited fruit rot. Epicuticular wax functions as a barrier to various environmental stresses in other fruit crops and may be a promising feature to mitigate sunscald in cranberry. In this study we assessed the function of epicuticular wax in cranberries to attenuate stresses associated with sunscald by subjecting high and low epicuticular wax cranberries to controlled desiccation and light/heat exposure. A cranberry population that segregates for epicuticular wax was phenotyped for epicuticular fruit wax levels and genotyped using GBS. Quantitative trait loci (QTL) analyses of these data identified a locus associated with epicuticular wax phenotype. A SNP marker was developed in the QTL region to be used for marker assisted selection.

**Results:**

Cranberries with high epicuticular wax lost less mass percent and maintained a lower surface temperature following heat/light and desiccation experiments as compared to fruit with low wax. QTL analysis identified a marker on chromosome 1 at position 38,782,094 bp associated with the epicuticular wax phenotype. Genotyping assays revealed that cranberry selections homozygous for a selected SNP have consistently high epicuticular wax scores. A candidate gene (*GL1-9*), associated with epicuticular wax synthesis, was also identified near this QTL region.

**Conclusions:**

Our results suggest that high cranberry epicuticular wax load may help reduce the effects of heat/light and water stress: two primary contributors to sunscald. Further, the molecular marker identified in this study can be used in marker assisted selection to screen cranberry seedlings for the potential to have high fruit epicuticular wax. This work serves to advance the genetic improvement of cranberry crops in the face of global climate change.

**Supplementary Information:**

The online version contains supplementary material available at 10.1186/s12870-023-04207-w.

## Introduction

As the climate changes, the cranberry industry will become increasingly strained by severe and unpredictable weather events and shifts in season length. The American cranberry (*Vaccinium macrocarpon*) is a climate sensitive fruit crop confined to acidic peat soils of temperate regions at latitudes of about 39 degrees in the northern hemisphere [1]. Similar to other temperate-adapted fruits such as apples, plums, and grapes, cranberries have a chilling requirement during their dormant period and require at least 62 days per year below 7 ℃ for adequate bloom and fruit set [2]. Shorter, milder winters challenge the achievement of these required low temperatures. Elevated and fluctuating temperatures in the spring induce early flower bud development which risks ecological mismatch in pollinator timing [3] and risk of sudden frost damage [4]. In the summer, prolonged heat waves and drought conditions promote sun damage to developing berries, a condition known as sunscald.

Sunscald results from a combination of excessive photosynthetically active radiation, UV radiation, high temperature, and can be worsened by low humidity [5]. The fruit injury due to sunscald can manifest in any of three modes: (1) heat injury sunscald, where fruits develop a cooked appearance, (2) ultraviolet radiation sunscald, a condition commonly found in fruits growing at high altitudes, and (3) photodynamic sunscald of heated tissues, which results from absorption of visible energy by photosensitive pigmented cells with temperature induced chemical lesions (Additional file 1) [6]. Environmental conditions associated with sunscald include clear skies, air temperatures of 27 ℃ or higher, canopy and/or mid-canopy temperatures of 36–41 ℃ [7]. Sunscald is difficult to avoid in cranberry since the cranberry plants have a low-lying shrubby growth habit and shade from surrounding plants is limited. Fruit in the upper part of the cranberry canopy can be fully exposed to the sun. Incidence of sunscald is common in the cranberry industry and has been documented in all commercial cranberry growing regions of the U.S. and Canada [8].

Conventional practices to prevent sunscald in fruit crops include sprinkler cooling systems, improved air movement through the field, avoiding excessive summer pruning, shade netting, and applying sunburn suppressants and protective coatings to fruit surfaces [9]. In cranberry, growers typically turn on irrigation systems when air temperature exceeds 27 ℃ [7]. Running the irrigation for 20 min was demonstrated to cool plants between 5–10.2 ℃ [7]; however, this method is accompanied by the disadvantages of increased water and power usage as well as potentially increased fruit rot. In addition, oversaturating roots can create ideal conditions for pathogenic fungi, such as root-rotting *Phytophthora* spp. [10, 11]. A possible alternative to controlling sunscald is to breed new cultivars with features that enhance resistance to heat, light, and desiccation stresses.

Cuticular wax and cutin are two components of the plant cuticle that forms a protective acellular layer surrounding plant organs. Cutin forms a matrix of mid chain hydroxy and epoxy C6 and C18 fatty acids. Cuticular wax is composed of a complex mixture of very long chain (C20-C40) fatty acids and their derivatives which include alkanes, aldehydes, primary and secondary metabolites, alcohols, ketones, and esters [12–16]. There are two layers of waxes on plant surfaces: (1) epicuticular wax (ECW), which forms highly crystalline structures on plant surfaces and establishes a continuous hydrophobic self-cleaning surface [17–19], and (2) intracuticular wax, which is embedded into the cutin matrix and functions to establish the transpirational barrier in plants [17, 20]. Several comprehensive reviews provide insight into the genetic cues governing cuticular wax synthesis and deposition [15, 21–23].

Plants dynamically regulate their cuticular wax load and chemical composition in response to water shortage, temperature, and excess heat—three environmental factors associated with sunscald [14, 24–26]. However, the function of ECW on heat/light and temperature stress in cranberries has yet to be tested. In addition to forming a barrier against abiotic environmental stress, the crystalline structure and biochemical components of cuticular wax may affect fungal pathogen adherence to the berry surface and subsequent infection. Sunscald causes degradation of the crystalline structure of ECW into amorphous masses, which leads to a higher permeability and dehydration [27]. Increased permeability risks enhanced penetration of fungal pathogens [28]. Secondary fungal infections following sunscald is a common occurrence in cranberries [29] as well as other fruits and vegetables [30–33]. Recent evidence suggests the resistance of cranberries to fungal infections that cause fruit rot is more related to how cranberries respond to fungal infection rather than the presence of certain fungal species [34]. Therefore, strengthening innate fortification features of cranberry, such as epicuticular wax, against environmental predispositions may prove a more effective approach to control cranberry pathogens.

Recent progress has been made to link priority cranberry traits with the genome [35]. Next generation sequencing methods have been used to assemble the cranberry plastid [36], mitochondria [37], and nuclear [38–40] genomes. The identification of quantitative trait loci (QTLs) can further pinpoint regions of the genome that harbor molecular variation governing traits of interest. To date, QTLs have been identified that are associated with cranberry fruit rot resistance, yield, berry weight, and titratable acidity [41, 42], fruit shape and size [43], anthocyanin content and other phytochemicals [44], and low citric and malic acid [45, 46]. Nucleotide variation identified within or near a given QTL can then be exploited for use in marker assisted selection. Marker assisted selection expedites the traditional breeding process by utilizing molecular markers associated with a targeted trait, so that plants can be genotyped as seedlings and only those predicted to exhibit the desired phenotypes are selected for further evaluation. The identification of molecular markers associated with ECW may be an effective approach to breed new cranberry cultivars with enhanced ECW, thus fortifying cranberry environmental defenses.

Cranberry epicuticular wax functions as a primary barrier between soft fruit tissues and the environment. Thus, it is important to investigate when seeking to protect fruits from environmental stress such as sun scald and secondary pathogen infection. Considering the protective functions and dynamic environmental responses of cuticular wax in other fleshy fruits, we hypothesize that high ECW may likewise attenuate the effects of environmental stresses (specifically heat, light, and water loss) in cranberry. The objectives of this study are to (1) test whether cranberry ECW mitigates effects of heat/light and desiccation, (2) utilize QTL analyses to identify regions of the genome associated with increased wax content, (3) generate a molecular marker and identify candidate gene(s) associated with ECW, and finally (4) validate generated markers using PCR allele competitive extension (PACE) genotyping assays. The completion of this work adds to the arsenal of molecular tools aiming to improve the sustainability of cranberry production.

## Results

### High epicuticular wax on cranberries imparts protection from heat and desiccation

To investigate the role of ECW in mitigating environmental stressors that contribute to loss of yield, cranberries with high and low ECW were subjected to controlled heat/light and desiccation experiments.

**Fig. 1:**
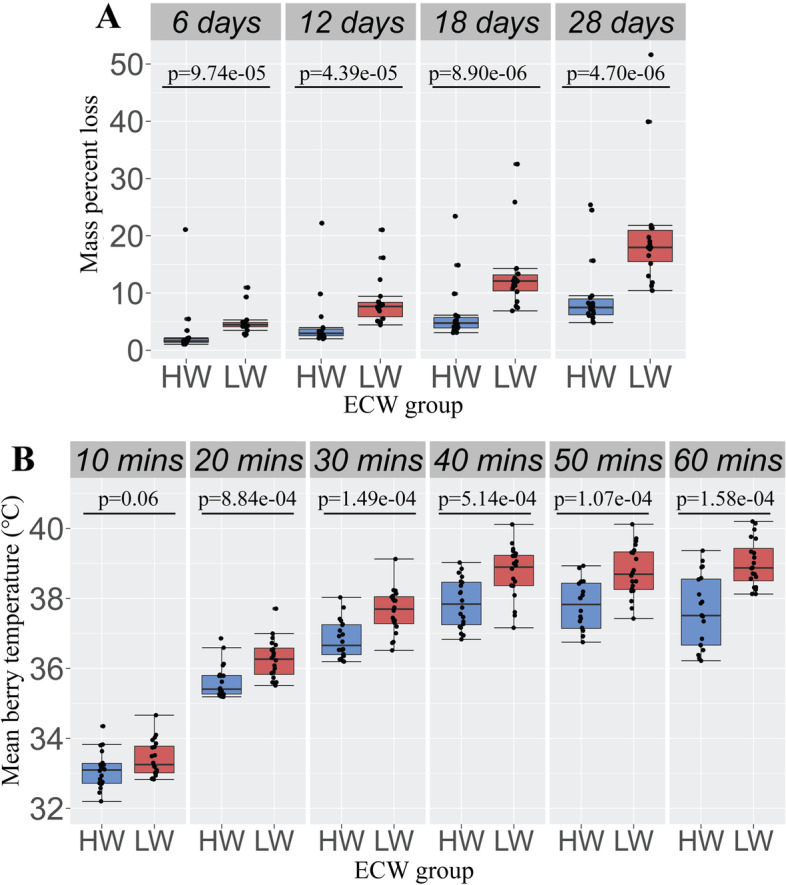
Cranberry epicuticular wax reduces water loss and berry surface temperature. **A** Following controlled desiccation in an incubator over 28 days, cranberries with low epicuticular wax (LW) lost a significantly greater mass percent than those with high epicuticular wax (HW). **B** HW and LW berries were irradiated with light for a period of 60 min and mean berry surface temperature was calculated for every 10 min. For all time points beyond t = 10 min, HW cranberries maintain a significantly lower estimated mean berry surface temperature than LW cranberries. All *p* values based on pairwise Tukey HSD tests

Following controlled desiccation in an incubator after 28 days, the mean berry mass percent loss at t = 28 days for low ECW berries was 20.2 ± 10.10 and for high ECW berries was 9.5 ± 6.11 (Table 1). Both berry wax load and time in the incubator (6, 12, 18 or 28 days) had an effect on berry mass percent loss (*p* = 3.53e-10, *p* = 5.50e-16, respectively; One-way analysis of variance (ANOVA; Table 2). At each time point, high ECW berries retained significantly greater mass than those with low ECW (6 days: *p* = 9.74e-05, 12 days: *p* = 4.39e-05, 18 days: *p* = 8.9e-06, 28 days: *p* = 4.70e-06; Tukey HSD; Fig. 1A; Table 3). This suggests that ECW may help protect cranberries from desiccation. There existed three data outliers for each time point; however, these data did not affect overall conclusions.

**Table 1:** Summary statistics for berry mass percent loss in high vs. low wax berries at each time point in controlled desiccation study

*time point*	*wax*^*a*^	*n*^*b*^	*mean (min, max)*^*c*^	*σ*^*d*^
6 days	HW	18	2.96 (1.06, 21.1)	4.64
	LW	18	4.84 (2.62, 11.0)	2.10
12 days	HW	18	4.47 (2.00, 22.2)	4.79
	LW	18	8.52 (4.43, 21.0)	4.21
18 days	HW	18	6.38 (3.07, 23.4)	5.10
	LW	18	13.1 (6.88, 32.5)	6.36
28 days	HW	18	9.50 (4.83, 25.4)	6.11
	LW	18	20.2 (10.4, 51.6)	10.10

Cranberries with high and low epicuticular wax were subjected to controlled desiccation in an incubator for 28 days. For each berry, mass (in grams) was measured at each time point (6,12,18, and 28 days), and mass percent loss was calculated

^a^wax– high wax (HW), low wax (LW)

^b^number of individuals per group

^c^mean, minimum, and maximum mass percent loss

^d^standard deviation

**Table 2:** One-way ANOVAs assessing the effect of high vs. low epicuticular wax and time on mean surface temperature and mass percent loss

*experiment*	*independent variable*		*response variable*	*df*^*b*^	*ss*^*c*^	*ms*^*d*^	*f*^*e*^	*p*^*f*^
desiccation		wax^a^ (HW/LW)	mass percent loss	1	4.47	4.47	45.53	3.53e-10
desiccation		time (days)	mass percent loss	3	7.56	2.52	32.45	5.50e-16
heat/light		wax (HW/LW)	mean surface temperature (°C)	1	313.20	313.23	17.48	4.08e-05
heat/light		time (mins)	mean surface temperature (°C)	5	3347.90	669.59	127.40	< 2.20e-16

^a^ wax– high wax (HW), low wax (LW)

^b^degrees of freedom

^c^sum of squares

^d^mean sum of squares

^e^f statistic of the ANOVA model

^f^*p* value associated with the f-statistic, significance defined as p ≤ 0.05

**Table 3:** Tukey HSD tests: detecting pairwise differences in transformed berry mass percent loss between high wax and low wax berries at each time point

*time point*	*difference*^*a*^	*lower*^*b*^	*upper*^*c*^	*p adjusted*^*d*^
6 days	0.36	0.19	0.53	9.74e-05
12 days	0.35	0.20	0.50	4.39e-05
18 days	0.35	0.22	0.49	8.90e-06
28 days	0.35	0.22	0.48	4.70e-06

^a^difference in the observed means

^b^lower end of the 95% confidence interval

^c^upper end of the 95% confidence interval

^d^adjusted *p* value after multiple comparisons, significance defined as p ≤ 0.05

Following controlled heat/light experiments, mean berry surface temperature at t = 60 min for low ECW berries was 39.0 ℃ ± 0.67 and for high ECW berries 37.6 ℃ ± 1.02 (Table 4). Wax load had a significant effect on mean berry temperature (*p* = 4.08e-05; One-way ANOVA; Fig. 1B; Table 4). For each time point greater than t = 10 min (10 min: *p* = 0.06), mean berry temperature of low ECW berries was greater than the mean berry temperature for high ECW berries (20 min: *p* = 8.84e-04, 30 min: *p* = 1.49e-04, 40 min: *p* = 5.14e-04, 50 min: *p* = 1.07e-04, 60 min *p* = 1.58e-05; Tukey HSD; Fig. 1B; Table 5). This suggests that cranberries with higher ECW are able to maintain a lower surface temperature when exposed to direct light stress than those with lower wax.

**Table 4:** Summary statistics for mean berry temperature in high vs. low wax berries at each time in heat/light experiment

*time point*	*wax*^*a*^	*n*^*b*^	*mean (min, max)*^*c*^	*σ*^*d*^
10 min	HW	20	33.1 (32.2, 34.4)	0.52
	LW	20	33.4 (32.8, 34.7)	0.51
20 min	HW	19	35.6 (35.2, 36.9)	0.48
	LW	20	36.3 (35.5, 37.7)	0.57
30 min	HW	20	36.8 (36.2, 38.0)	0.54
	LW	20	37.6 (36.5, 39.1)	0.63
40 min	HW	20	37.9 (36.8, 39.0)	0.70
	LW	20	38.7 (37.2, 40.1)	0.75
50 min	HW	19	37.8 (36.8, 38.9)	0.69
	LW	20	38.8 (37.4, 40.1)	0.73
60 min	HW	20	37.6 (36.2, 39.4)	1.02
	LW	20	39.0 (38.1, 40.2)	0.67

High wax (HW) and low wax (LW) cranberries were subjected to controlled application of heat and light for t = 60 min total. Mean berry surface temperature was measured at each time point (10,20,30,40,50, and 60 min)

^a^wax– high wax (HW), low wax (LW)

^b^number of individuals per group

^c^mean, minimum, and maximum temperature in °C

^d^standard deviation

**Table 5:** Tukey HSD tests: detecting pairwise differences in transformed berry mean surface temperature between high wax and low wax berries at each time point

*time point*	***difference***^***a***^	***lower***^***b***^	***upper***^***c***^	***p adjusted***^***d***^
10 min	0.34	-0.02	0.69	0.06
20 min	1.22	0.53	1.91	8.84e-04
30 min	2.07	1.07	3.06	1.49e-04
40 min	2.76	1.29	4.22	5.14e-04
50 min	3.11	1.65	4.57	1.07e-04
60 min	4.22	2.49	5.94	1.58e-05

^a^Difference in the observed means

^b^Lower end of the 95% confidence interval

^c^Upper end of the 95% confidence interval

^d^Adjusted *p* value after multiple comparisons, significance defined as *p* ≤ 0.05

### QTL Analysis identifies a locus and candidate genes associated with epicuticular wax in cranberry

**Fig. 2:**
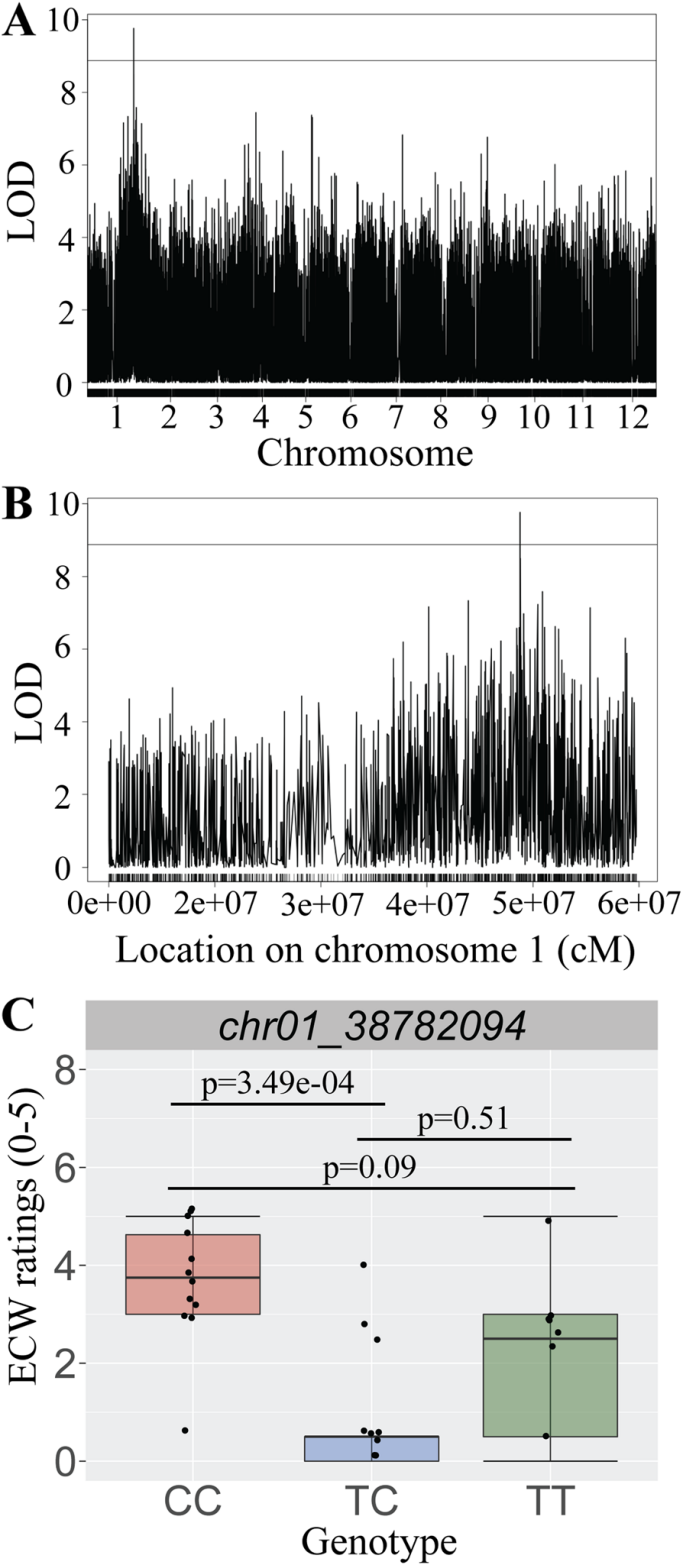
QTL region associated with epicuticular wax and a SNP assay identifies consistently predicts epicuticular wax scores. **A** A QTL associated with the epicuticular wax phenotype was identified on chromosome 1 of the cranberry genome. The threshold (1000 permutations) was identified to be LOD 8.88. **B** On chromosome 1, the bounds of the QTL consist of the peak at position 38,782,094 (LOD 9.76), and the ends of the QTL interval markers at positions 38,782,027 and 38,878,190. **C** A subset of cranberry progeny was selected for marker validation using PCR allele competitive extension (PACE) genotyping assays. Assay results revealed that individuals that are homozygous CC have consistently high wax ratings (with the exception of one outlier). The majority of individuals that are homozygous TT have low wax ratings; however, there is more within group variation than homozygous CC. Error bars represent standard error. All *p* values based on pairwise Dunn’s tests

A population segregating for ECW level was genotyped using genotyping by sequencing (GBS). A total of 27,790 potential SNP markers was used in QTL analysis. A single peak was identified with an interval covering 96 kbp on chromosome 1, at position 38,782,094, with a LOD score of 9.76 (threshold 8.88 at 5%; Fig. 2A-B). This QTL was calculated to account for 36.2% of the variability of the observed wax phenotype. A single nucleotide polymorphism (SNP) was identified with a LOD of 9.76 and was used for SNP genotyping assay design. Within the QTL interval, there were 11 potential gene sites identified. Additionally, candidate genes identified within 1 MB of the QTL region include: a homolog of Glossy1 (GL1-9; position = 37,769,360–37,769,360 bp), and a homolog of SAR1A (position = 38,883,064–38,883,064 bp). Fragments of SUR2 were also found (position = 37,772,215–37,772,763 bp), as well as SYP45 (position = 38,691,169–38,691,456 bp). Among these, GL1-9 is the most likely to be directly associated with ECW.

### Epicuticular wax phenotypes can be predicted from genotyping assays

A PACE genotyping assay was designed for a marker identified on chromosome 1, position 38,782,094 bp (chr01_ 38,782,094) and was used for genotyping cranberry accessions varying in ECW. Among the subset of 34 CNJ15-55 individuals chosen for high, medium, and low ECW phenotypes, for marker chr01_38782094, there were *n* = 12 (35.3%) homozygous CC individuals, *n* = 13 (38.2%) homozygous TT individuals, and 9 (26.5%) heterozygous TC individuals. Genotyping results demonstrate that for this marker, there exist significant differences in wax ratings among genotypes (*p* = 8.12e-07; Kruskal–Wallis test). Individuals that are homozygous CC had consistently high ECW ratings (Fig. 2C), with the exception of one outlier. Individuals homozygous CC had significantly higher ECW phenotypes than those homozygous TT (chr01_38782094: *p* = 3.49e-04; Dunn’s test). However, heterozygous TC individuals did not significantly differ in ECW wax ratings from those homozygous CC (*p* = 0.09; Dunn’s test) nor those homozygous TT (*p* = 0.51; Dunn’s test). These results suggest that while this marker can be used to reliably predict high or low ECW phenotypes of cranberry seedlings (i.e. homozygous CC vs. homozygous TT), phenotypic predictions for individuals that are heterozygous TC are not so easily discerned.

## Discussion

### Increased epicuticular wax mitigates heat and desiccation stresses

Here we test the hypothesis that cranberry ECW plays a role in controlling surface temperature and water loss. Our results suggest that berries with high ECW can maintain a lower temperature upon direct light irradiation and lose less mass when subjected to controlled desiccation in an incubator.

Much of the light reflectance and water permeance of ECW is a function of the ECW crystallinity, which is influenced by its biochemistry. The presence of long-chain alkanes and aldehydes promotes the formation of crystalline, water impermeable ECW, where triterpenoids and sterols had opposite effects [47–54]. In rice, disorganized wax crystals of the wilted dwarf and lethal 1 (*wdl1*) mutant lead to a 2.3 times increased transpiration rate than wild type and increased water loss and lower water use efficiency [55].

The light, powdery characteristics of cranberry ECW may function to block damaging light irradiation by scattering excess light before it can be absorbed into the fruit tissues causing sun damage. The degree of light reflectance is largely affected by the size, distribution, and orientation of ECW crystals, where plate-like crystals are more favorable for reflecting light than amorphous wax [56]. Thicker ECW also has the capacity to reflect more light. Sun exposed grape berries with higher ECW load reflect 10–70% more incoming radiation than shaded berries with lower ECW load [56]. Amorphous ECW also has increased water permeance and likelihood for dehydration when compared to crystalline ECW [27]. The color of fruit is also known to affect temperature, and exposed dark colored berries measure up to 5 ℃ higher surface temperature than exposed light colored berries [5, 57]. A cranberry variety known for dark fruit color, ‘Early Black’, is cited as being more prone to sunscald than the lighter-colored ‘Stevens’ and ‘Ben Lear’[8].

Plants actively regulate their cuticle in response to environmental stress such as water deficit and light irradiation, which provides plants the opportunity to dynamically optimize their primary barrier to enhance survival [52, 58–61]. Fruits from plants adapted to water deficit conditions often have well-developed cuticles [26, 62–66]. Conversely, conditions of high humidity decrease cuticle deposition [64]. Increased light irradiation increases the thickness of cuticular wax in many plants [64, 67, 68]. For example, light with enhanced UV-B and UV-C was shown to increase cuticular wax load, particularly by increasing the alkane fraction [64]. ECW has also been demonstrated to reflect UV-B and UV-C light while absorbing photosynthetic light protecting plant cells from harmful irradiation [69, 70].

In cucumber, the expression of fruit specific wax biosynthesis genes *CsCER1* and *CsWAX2* increases under drought and salinity stresses [65, 66]. In tomato, regulation of SISHN1 transcription factor induces expression of wax biosynthesis genes leading to enhanced cuticular wax deposition and drought tolerance compared to control plants [71]. Studies also suggest that chemical composition of the wax rather than overall wax load is potentially more influential in promoting drought tolerance [48, 72, 73].

### QTL analysis gives insight into a gene candidate for targeting cuticular wax synthesis in cranberry

Within the QTL interval, 11 potential genes were identified by comparison to the reference genome. Most of the 11 were genes of unknown function or genes that are not known to be relevant to wax production. Expanding the search for candidate genes, within 1 Mb of the QTL, allowed the identification of three potential gene candidates: *Glossy 1–9* (*GL1-9*), *SAR1A*, and *SYP43*.

Although *SAR1A* and *SYP43* are not directly related to ECW biosynthesis, they have been associated with cellular pathways leading to ECW secretion. *SAR1A* functions in mediating protein export from the endoplasmic reticulum [74] and is found to be upregulated during periods of osmotic stress and high wax deposition [75]. *SYP43* is associated with golgi stability during stress [76–78], which is a key component to wax secretion [79]. *SYP43* also relates to fungal host resistance as shown with powdery mildew [76–78].

The identification of *GL1-9* in cranberry and its association with ECW and drought tolerance is promising for future efforts to mitigate sunscald. However, sequence analysis of the promoter region of GL1-9 showed no consensus variation between the high wax and low wax groups (Additional file 3). There are likely still unidentified genes or promoter sequences that harbor the causative variation among candidate genes. The current resolution of the QTL analysis limits reasonable exploration of the causative variation in this region.

Glossy (GL) of *Zea mays* and ECERIFERUM (CER) of *Arabidopsis thaliana* are two notable gene families associated with wax synthesis. *CER1*, *CER2*, *CER3*, *CER6*, *GL1*, and *GL8* encode wax synthesis-related proteins and enzymes involved in the transport of wax compounds [80–85]; whereas, *GL2*, *GL15* encode wax regulatory proteins [86–89]. *GL15* is a developmental gene found to regulate epidermal cell identity in maize [87, 90, 91]. A recessive mutation in gl15 plants switches the plant epidermis from juvenile to adult stage earlier than wild-type. Mutant *gl15* plants lack visible ECW, have epidermal hairs, rectangular epidermal cells, and highly crenulated lateral walls [90–92]. *GL* analog genes have also been found in rice. Overexpression of *GL1-2* in *Oryza sativa* induced more wax crystallization and a thicker epicuticular wax layer, while the mutant had less wax crystallization and a thinner cuticular layer [93]. Further, the *osgl1-2* mutant is more sensitive to drought stress at the reproductive stage, which suggests that *OsGL1-2* is important for conferring drought resistance [93].

The 11 homologous genes of *GL1* are closely related to the *CER1* gene in *Arabidopsis thaliana* (*AtCER1*) [93]. *CER1* encodes an enzyme involved in the decarbonylation pathway of epidermal wax synthesis. Within this pathway, *CER1* and *CER3* act together to catalyze the formation of alkanes from VLCFA-CoA [80, 94]. *CsCER1* (*CER1*) and *CsWAX2* (*CER3*) of cucumber, *PaCER1* of sweet cherry, were also demonstrated to play important roles in very long chain alkane biosynthesis and drought tolerance [26, 65, 66, 94]. In yeast, heterologous expression of the combination of *CER1*, *CER3*, a cytochrome b5, and *LACS1* resulted in the formation of very long chain alkanes [95]. *LACS1* is required for C16 cutin biosynthesis and is required for the normal accumulation of downstream wax compounds [96]. *WSL2* in rice was demonstrated to encode a homolog of *CER3* of *Arabidopsis thaliana* and *GL1* of *Zea mays* [97]. Mutants produced a reduced quantity of very long chain fatty acids suggesting that *WSL2* is associated with fatty acid elongation [97].

The chemical composition and the quantity/proportion of cuticular wax primarily governs the environmental function of the plant cuticle and exhibits high variability between plant species, plant organs, and even different plant developmental stages [98, 99]. Approximately 50% of cranberry cuticular wax is composed of triterpenoids [100], which are secondary metabolites that have anti-cancer, anti-inflammatory, antimicrobial, and cardioprotective properties in humans [101, 102]. Ursolic acid, a triterpenoid abundant in cuticular wax of cranberries, has been found to suppress *Alternaria alternata* in apple [103], inhibit spore germination of *Aschochyta raibiei* on chickpea [104] and grain mold fungi on sorghum [105]. However, it is unknown whether ursolic acid has antifungal activities in cranberries. Fungi that cause fruit rot in cranberry induce disease in mature fruit tissues by secretion of ROS into fruit, resulting in a cascade of events that leads to cell death and fruit rot [106, 107]. Benzoic and quinic organic acids have been shown to reduce growth and ROS secretion by fruit rotting fungi [107]. Modifying the chemical composition of cuticular wax may also be an effective approach to combating fungal pathogens in cranberry. Conversely, there is evidence that epicuticular wax may facilitate fungal infection and growth [108–112]. The role of cuticular wax in fungal pathogenicity likely depends upon a combination of the presence of antifungal wax constituents and favorable environmental conditions which further induce wax constituent modification. More research is needed to understand the relationship between cuticular wax composition, biosynthesis and fungal pathogenicity in cranberries.

## Conclusions

Here we tested the biological roles of cranberry fruit ECW and used next generation sequencing tools and QTL analyses to identify genetic regions associated with ECW. Our results demonstrate that cranberries with high ECW can maintain a cooler surface temperature and slow berry water loss compared to those with low ECW. Within the QTL, a SNP marker was identified that associated with epicuticular wax level. We will utilize the identified marker in marker assisted selection to breed new varieties of cranberries with potentially increased resistance to heat stress. Finally, we have identified a gene candidate near the QTL region involved in alkane biosynthesis of cuticular wax.

## Materials and methods

### Plant material and phenotyping

**Fig. 3:**
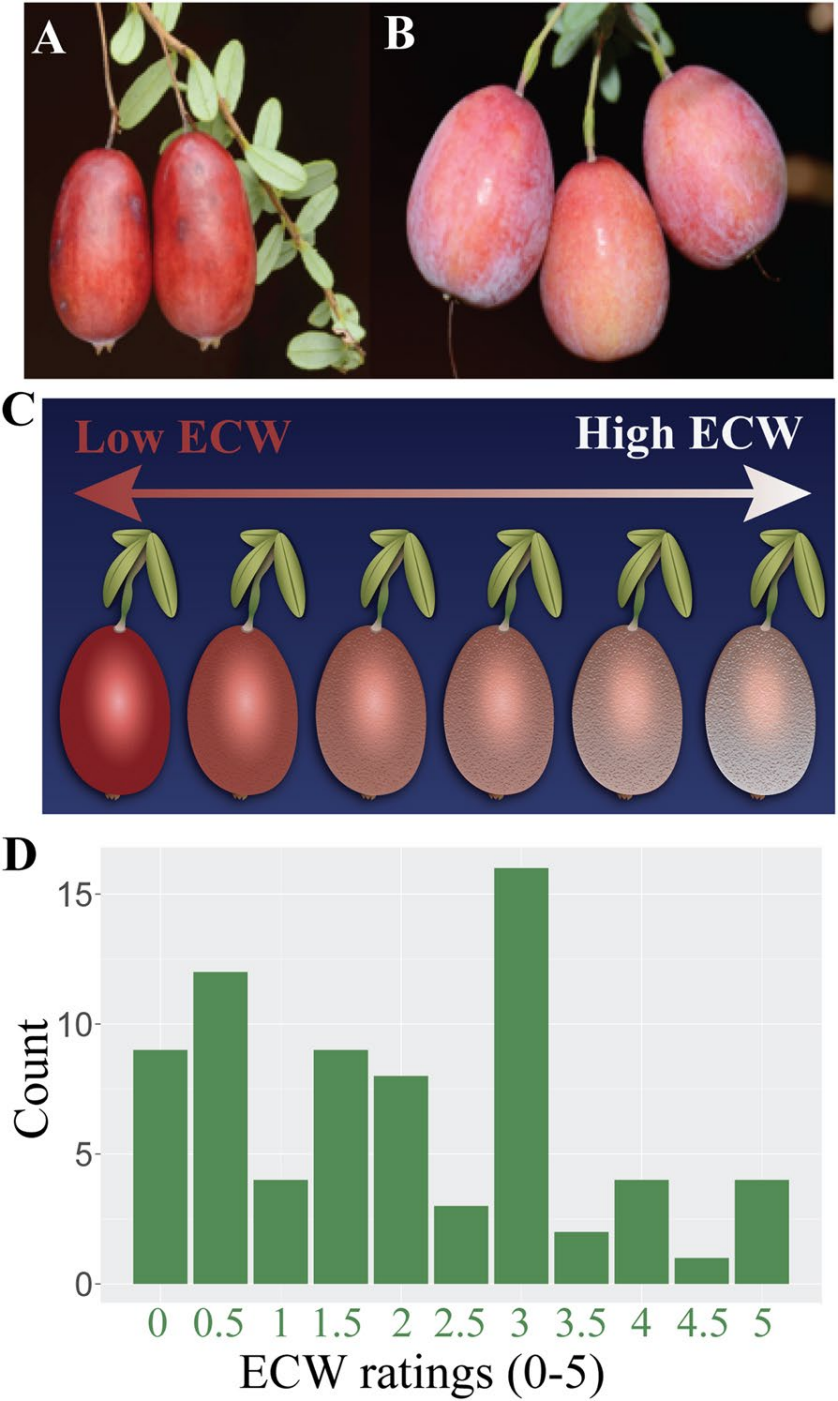
Phenotyping system for cranberry cuticular wax. **A** Cranberries with low epicuticular wax (ECW) have a shiny, smooth appearance and **B** those with high ECW have a dull, white, powdery surface appearance. **C** Cranberry ECW phenotyping was conducted manually using a subjective visual rating system of 0–5 corresponding to the relative amount of ECW load on the surface of berries, where 0 denotes little wax and 5 denotes relatively high ECW. **D** Frequency distribution illustrates the variability in ECW rating within the QTL population (*n* = 68)

Ninety individuals from a cranberry population (CNJ15-55) which segregates for ECW, were used for this study. CNJ15-55 was derived from a cross between CNJ08-103–20 and CNJ11-45–23 made in May 2015 (Additional file 2). The cross and subsequent population development are part of the ongoing cranberry breeding program, directed by N. Vorsa, at the P.E. Marucci Center for Blueberry and Cranberry Research and Extension, Chatsworth, NJ**.** We confirm that our study protocol complies with relevant institutional, national, and international guidelines and legislation. CNJ15-55 individuals were seeded in December 2015, transplanted into 10 cm square pots in June 2016, and maintained in a greenhouse at the P.E. Marucci Center for Blueberry and Cranberry Research and Extension. At time of transplanting, plants received 1.4 g fertilizer prills (Osmocote 14–14-14). In subsequent years, plants were fertilized biweekly from late May to mid-August with 20–20-20 soluble fertilizer at 185 ppm. Plants were watered daily during growing season (April–October) and weekly during dormancy (November-March). The average greenhouse temperature from April through October, was 75°F, and during dormancy, 40°F. To achieve pollination and fruit set, plants were placed outside during flowering. High ECW cranberries are characterized by the appearance of a dull, white powdery surface (Fig. 3A-B). For all plants that set fruit (*n* = 73), fruit ECW was visually rated in September 2018 on a scale between 0–5, with 0 being the least amount of wax and 5 the highest wax rating (Fig. 3C-D**; **Table 6).

**Table 6:** Summary of epicuticular wax ratings in CNJ15-55

*rating*^*a*^	*overall (N* = *68)*^*b*^
0	9 (12.5%)
0.5	12 (16.7%)
1	4 (5.6%)
1.5	9 (12.5%)
2	8 (11.1%)
2.5	3 (4.2%)
3	16 (22.2%)
3.5	2 (2.8%)
4	4 (5.6%)
4.5	1 (1.4%)
5	4 (5.6%)

^a^Cranberry fruit epicuticular wax was visually rated on a scale of 0–5, with 0 being the least amount of wax and 5 the highest wax rating

^b^number of plants and percentage of plants with each wax phenotype

### Cranberry desiccation

A total of 18 cranberry fruit with high ECW and 18 cranberry fruit with low ECW were initially weighed and placed in an incubator (Thermo Fisher Scientific, Waltham MA, USA) at 30 °C for a period of 28 days. Berries were re-weighed at 6, 12, 18, and 28 days after the experiment began and mass percent loss was calculated at each time point.

### Heat/light application

**Fig. 4:**
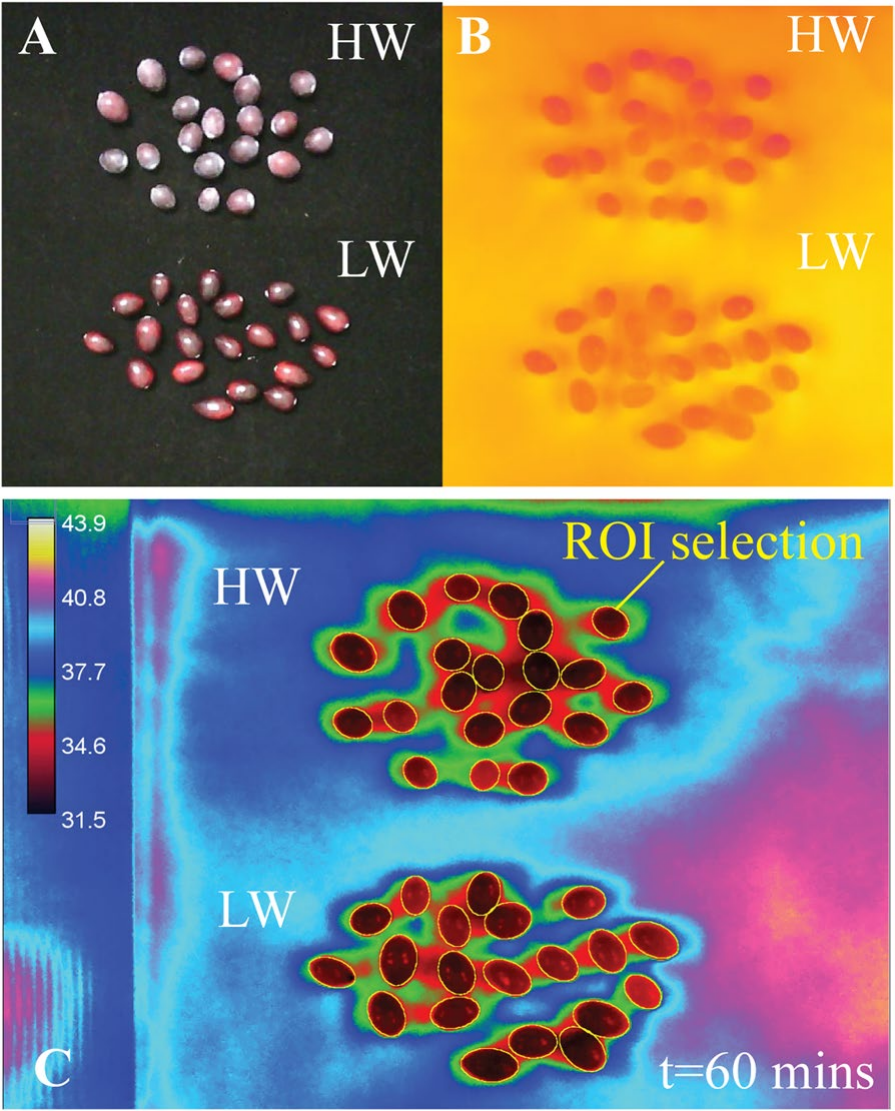
FLIR image capture and temperature calculation using Fiji and ThermimageJ. **A** Cranberries with high epicuticular wax (top) and low epicuticular wax (bottom) were irradiated with direct light for a period of 60 min. **B** Raw images were captured using a FLIR camera every minute and cranberry surface temperature was measured every 10 min. **C** Using ThermimageJ macros in Fiji, thermal images were re-colored with the LUT tool. The area of each berry was selected as a region of interest and mean, min & max temperatures were calculated. Images B and C represent berries at time point t = 60 min of light irradiation

A total of 20 individual cranberry fruit with high ECW and 20 with low ECW (Fig. 4A) were irradiated with 4 Bogen Quartz light heads (250 W; Bogen Photo Corp., Fairlawn NJ, USA) for a total of 60 min. Using a quantum meter (Model MQ-200, Apogee instruments, Logan UT, USA), light intensity for both high and low wax experimental groups was measured to be an average of 54.4 ± 3.82 µmol m^−2^ s^−1^, where differences in intensity between groups were not statistically significant (*p* = 0.9, one-way ANOVA). Infrared thermal images were captured once per minute using a forward looking infrared (FLIR) A70 camera (Teledyne FLIR LLC, Wilsonville OR, USA). Thermal image jpegs captured at time points 10, 20, 30, 40, 50, and 60 min of irradiation were imported into Fiji image analysis software [113] for raw data temperature extraction using the ThermimageJ macro [114] **(**Fig. 4B). *LUT* > *6 shades* was selected for thermal image color range (Fig. 4C). At each time point, the area of each berry was selected using the ellipses tool (Fig. 4C) and added to the region of interest (ROI) manager. In the *Analyze* > *set measurements* function, measurement parameters selected included *mean gray value*, *standard deviation*, and *min & max gray value*. Estimated temperature values are embedded in each pixel of infrared thermal images; therefore, the *mean gray value* of each berry outputs the mean temperature of that berry. Finally, the *Analyze* > *Measure* function outputted the mean, min, and max temperature for each berry.

### DNA extraction

DNA was extracted from leaf tissue using a modified CTAB protocol and GBS libraries were generated as in Daverdin et al. [42]. The GBS libraries were prepared using Msp1 and Pst1 as the restriction enzymes (New England Biolabs Inc., Ipswich, Massachusetts, USA). Prepared GBS libraries were sent to Genewiz, Inc. (South Plainfield, NJ, USA) for sequencing on the Illumina Hi-seq platform (Illumina Inc., San Diego, California, USA) with a 2 × 150 bp paired-end configuration.

### QTL analysis

Barcoded samples were de-multiplexed using STACKS and aligned to the cranberry reference genome [40]; Size:488 Mbp, Contigs:124, N50: 15.4 Mbp) with bwa-mem [115, 116]. Samtools was used to call SNPs [117]. SNP filtering and QTL analysis was performed as in Fong et al. [46], where qualifying SNPs required a read support of at least 4 reads, and heterogeneity between 25 and 75%. Missing data were limited to only 10% of the population for a given marker, and markers that were homogenous throughout the population were also removed. Markers were anchored according to their genetic positions and R/qtl was used to calculate genetic distance between markers and identify QTL [118]. Genome wide significance of LOD scores was calculated at *p* < 0.05 through 1000 permutations. Candidate genes in the QTL region were identified based on the current cranberry reference genome (https://www.vaccinium.org/bio_data/1254430). Potential candidate genes were otherwise identified through NCBI tblastx, where sequences from the KEGG cutin, suberin and wax biosynthesis—*Arabidopsis thaliana* (thale cress) pathway (ath00073)—were referenced against the cranberry reference genome, and locations noted in relation to the QTL region.

### Sequence analysis of candidate gene GL1-9

The GL1-9 reference sequence was used as annotated in the current cranberry reference genome sequence [40]. Primers were designed to amplify about 500 bp upstream of the start codon. Five representative accessions with high ECW and 5 representative accessions with low ECW accessions were amplified and sequenced. Amplicon quality was verified using Qiaxcel Screengel (Qiagen Sciences, Germantown MD, USA). Fragments were purified with EXOSAP-IT (Thermo Fisher Scientific, Waltham MA, USA). Sequencing was conducted using a Big Dye 3.1 kit (Thermo Fisher Scientific, Waltham MA, USA) and analyzed on an Applied Biosystems Genetic Analyzer 3500. Sequences were aligned to the GL1-9 reference sequence using BWA [116] and visualized in IGV [119].

### Marker validation

Using the genotypes generated from GBS in concert with the QTL associated with surface wax on chromosome 1, we identified a single-nucleotide polymorphism (SNP) at location 38,782,094 bp. FASTA formatted sequences for each SNP were sent to Integrated DNA Technologies (Coralville, IA, USA) and 3CR Bioscience (Harlow, Essex CM20 2BU, UK) for PCR allele competitive extension (PACE) primer pair design and manufacture. To validate the efficacy of markers, leaf tissue was collected from a total of *n* = 34 progeny from the CNJ15-55 population, including *n* = 7 with high surface wax (wax score = 4–5), *n* = 10 with medium surface wax (wax score: 3), and *n* = 17 with low surface wax (wax score = 0). DNA was extracted from leaf tissue using the modified CTAB procedure [42] The cyclic conditions for PACE genotyping reactions were conducted as follows: Enzyme activation at 94℃ for 15 min, followed by 10 cycles of template denaturation at 94℃ for 20 s and annealing and extension at 65–57℃ for 60 s dropping 0.8℃ per cycle, followed by 30 cycles of denaturation at 94℃ for 20 s and annealing and extension at 57℃ for 60 s.

### Statistical analysis

For desiccation and heat/light experiments, all statistical analyses and graph generation was performed using R ver. 1.4.2 [120] and figures were constructed using the *ggplot2* visualization package ver. 3.3.5 [121]. Normality was determined qualitatively using quantile–quantile plots and quantitatively using Shapiro–Wilk normality tests (*p* > 0.05) with default parameters. Residuals were checked for normality using Kolmogorov–Smirnov tests using function *ols_test_normality* of the *olsrr* package ver. 0.5.3 [122]. Both percent mass and average temperature deviated from normality, so data were transformed using Tukey’s ladder of powers function (function *transformtukey* within the *rcompanion* package ver. 2.4.13 [123]. We tested the hypothesis that differences exist in percent loss and mean temperature between cranberries with high ECW and low ECW using one-way analysis of variance (ANOVAs); function *aov* in *stats* package ver. 4.1.2; [120]. Pairwise differences were detected using Tukey’s honestly significant difference (function *TukeyHSD* in *agricolae* package ver. 1.3.5) [124]. The effect of marker genotype on wax phenotype was determined using non-parametric Kruskal–Wallis rank sum tests (function *kruskal.test* in *stats* package ver. 4.1.2) [120]. Pairwise differences in wax phenotype between genotypes was calculated using non parametric Dunn’s tests [125] with Bonferroni correction for p values (function *dunnTest* in *FSA* package ver. 0.9.3) [126]. Significance for all tests were defined as *p* ≤ 0.05.

## Supplementary Information

**Additional file 1: Figure S1.** Examples of sound and sunscald cranberries in a field setting.

**Additional file 2: Figure S2.** Pedigree for CNJ15-55, a cranberry population that segregates for epicuticular wax. Cranberry population CNJ15-55 was derived from a cross between CNJ08-103-20 and CNJ11-45-23 made in May 2015. Individuals within the CNJ15-55 population that set fruit were phenotyped for epicuticular wax and used in QTL analysis. Among these, a subset of 34 progeny with relatively high and low epicuticular wax was used for marker validation.

**Additional file 3.** 

## Data Availability

All data sheets and codes to process data are available upon request to the corresponding author, James Polashock (james.polashock@usda.gov). Sequence data generated for this study is available publicly in the NCBI Sequence Read Archive under BioProject ID: PRJNA888050.

## Abbreviations

ECW: Epicuticular wax
GL: Glossy
CER: ECERIFERUM

## References

[1] Vander Kloet SP. The genus *Vaccinium* in North America. Agriculture Canada; 1988.

[2] Gareau BJ, Huang X, Pisani Gareau T, DiDonato S (2020). The strength of green ties: Massachusetts cranberry grower social networks and effects on climate change attitudes and action. Clim Change.

[3] Ellwood ER, Playfair SR, Polgar CA, Primack RB (2014). Cranberry flowering times and climate change in southern Massachusetts. Int J Biometeorol.

[4] Gareau BJ, Huang X, Gareau TP (2018). Social and ecological conditions of cranberry production and climate change attitudes in New England. PLoS ONE.

[5] Gambetta JM, Holzapfel BP, Stoll M, Friedel M (2021). Sunburn in Grapes: a review. Front Plant Sci.

[6] Barber HN, Sharpe PJH (1971). Genetics and physiology of sunscald of fruits. Agric Meteorol.

[7] Pelletier V, Pepin S, Gallichand J, Caron J (2016). Reducing cranberry heat stress and midday depression with evaporative cooling. Sci Hortic.

[8] Croft PJ (1995). Field conditions associated with cranberry scald. HortScience.

[9] Lal N, Sahu N (2017). Management strategies of sun burn in fruit crops-A review. Int J Curr Microbiol Appl Sci.

[10] Polashock JJ, Vaiciunas J, Oudemans PV (2005). Identification of a new *Phytophthora* species causing root and runner rot of cranberry in New Jersey. Phytopathology.

[11] Polashock JJ, Caruso FL, Averill AL, Schilder AC (2017). Compendium of blueberry, cranberry, and lingonberry diseases and pests.

[12] Kunst L, Samuels AL (2003). Biosynthesis and secretion of plant cuticular wax. Prog Lipid Res.

[13] Pollard M, Beisson F, Li Y, Ohlrogge JB (2008). Building lipid barriers: biosynthesis of cutin and suberin. Trends Plant Sci.

[14] Samuels L, Kunst L, Jetter R (2008). Sealing plant surfaces: cuticular wax formation by epidermal cells. Annu Rev Plant Biol.

[15] Yeats TH, Rose JKC (2013). The formation and function of plant cuticles. Plant Physiol.

[16] Serrano M, Coluccia F, Torres M, L’Haridon F, Métraux J-P (2014). The cuticle and plant defense to pathogens. Front Plant Sci.

[17] Zeisler-Diehl V, Müller Y, Schreiber L (2018). Epicuticular wax on leaf cuticles does not establish the transpiration barrier, which is essentially formed by intracuticular wax. J Plant Physiol.

[18] Neinhuis C, Barthlott W (1997). Characterization and distribution of water-repellent, self-cleaning plant surfaces. Ann Bot.

[19] Schönherr J (1976). Water permeability of isolated cuticular membranes: The effect of cuticular waxes on diffusion of water. Planta.

[20] Zhang Y, Du Z, Han Y, Chen X, Kong X, Sun W (2020). Plasticity of the cuticular transpiration barrier in response to water shortage and resupply in *Camellia sinensis*: A role of cuticular waxes. Front Plant Sci.

[21] Post-Beittenmiller D (1996). Biochemistry and molecular biology of wax production in plants. Annu Rev Plant Physiol Plant Mol Biol.

[22] Hen-Avivi S, Lashbrooke J, Costa F, Aharoni A (2014). Scratching the surface: genetic regulation of cuticle assembly in fleshy fruit. J Exp Bot.

[23] Trivedi P, Nguyen N, Hykkerud AL, Häggman H, Martinussen I, Jaakola L (2019). Developmental and environmental regulation of cuticular wax biosynthesis in fleshy fruits. Front Plant Sci.

[24] Geyer U, Schönherr J (1990). The effect of the environment on the permeability and composition of *Citrus* leaf cuticles. Planta.

[25] Charles MT, Makhlouf J, Arul J (2008). Physiological basis of UV-C induced resistance to *Botrytis*
*cinerea* in tomato fruit: II. Modification of fruit surface and changes in fungal colonization. Postharvest Biol Technol.

[26] Xue D, Zhang X, Lu X, Chen G, Chen Z-H (2017). Molecular and evolutionary mechanisms of cuticular wax for plant drought tolerance. Front Plant Sci.

[27] Bondada B, Keller M (2012). Morphoanatomical symptomatology and osmotic behavior of grape berry shrivel. J Am Soc Hortic Sci.

[28] Ziv C, Zhao Z, Gao YG, Xia Y (2018). Multifunctional roles of plant cuticle during plant-pathogen interactions. Front Plant Sci.

[29] Akiva, Planche, Roy, Dana. AI on the bog: monitoring and evaluating cranberry crop risk. Proc Estonian Acad Sci Biol Ecol. 2021.

[30] Maughan T, Drost D, Black B, Day S. Using shade for fruit and vegetable production. All Current Publications. 2017. Paper 1654. https://digitalcommons.usu.edu/extension_curall/1654.

[31] McColloch LP, Cook HT, Wright WR (1982). Market diseases of tomatoes, peppers, and eggplant.

[32] Munné-Bosch S, Vincent C (2019). Physiological mechanisms underlying fruit sunburn. CRC Crit Rev Plant Sci.

[33] Kennelly M. Tomato Leaf and Fruit Diseases and Disorders: Diseases in Outdoor Production. Kansas State University. 2006. https://www.bookstore.ksre.ksu.edu/pubs/L721.pdf.

[34] Tadych M, Bergen MS, Johnson-Cicalese J, Polashock JJ, Vorsa N, White JF (2012). Endophytic and pathogenic fungi of developing cranberry ovaries from flower to mature fruit: diversity and succession. Fungal Divers.

[35] Gallardo RK, Klingthong P, Zhang Q, Polashock J, Atucha A, Zalapa J (2018). Breeding trait priorities of the cranberry industry in the United States and Canada. HortScience.

[36] Fajardo D, Senalik D, Ames M, Zhu H, Steffan SA, Harbut R (2013). Complete plastid genome sequence of *Vaccinium macrocarpon*: structure, gene content, and rearrangements revealed by next generation sequencing. Tree Genet Genomes.

[37] Fajardo D, Schlautman B, Steffan S, Polashock J, Vorsa N, Zalapa J (2014). The American cranberry mitochondrial genome reveals the presence of selenocysteine (tRNA-Sec and SECIS) insertion machinery in land plants. Gene.

[38] Polashock J, Zelzion E, Fajardo D, Zalapa J, Georgi L, Bhattacharya D (2014). The American cranberry: first insights into the whole genome of a species adapted to bog habitat. BMC Plant Biol.

[39] Diaz-Garcia L, Garcia-Ortega LF, González-Rodríguez M, Delaye L, Iorizzo M, Zalapa J (2021). Chromosome-level genome assembly of the American cranberry (*Vaccinium*
*macrocarpon* Ait.) and its wild relative *Vaccinium*
*microcarpum*. Front Plant Sci.

[40] Kawash J, Colt K, Hartwick NT, Abramson BW, Vorsa N, Polashock JJ (2022). Contrasting a reference cranberry genome to a crop wild relative provides insights into adaptation, domestication, and breeding. PLoS ONE.

[41] Georgi L, Johnson-Cicalese J, Honig J, Das SP, Rajah VD, Bhattacharya D (2013). The first genetic map of the American cranberry: exploration of synteny conservation and quantitative trait loci. Theor Appl Genet.

[42] Daverdin G, Johnson-Cicalese J, Zalapa J, Vorsa N, Polashock J (2017). Identification and mapping of fruit rot resistance QTL in American cranberry using GBS. Mol Breed.

[43] Diaz-Garcia L, Covarrubias-Pazaran G, Schlautman B, Grygleski E, Zalapa J (2018). Image-based phenotyping for identification of QTL determining fruit shape and size in American cranberry (*Vaccinium*
*macrocarpon* L.*)*. PeerJ.

[44] Diaz-Garcia L, Schlautman B, Covarrubias-Pazaran G, Maule A, Johnson-Cicalese J, Grygleski E (2018). Massive phenotyping of multiple cranberry populations reveals novel QTLs for fruit anthocyanin content and other important chemical traits. Mol Genet Genomics.

[45] Fong SK, Kawash J, Wang Y, Johnson-Cicalese J, Polashock J, Vorsa N (2020). A low citric acid trait in cranberry: genetics and molecular mapping of a locus impacting fruit acidity. Tree Genet Genomes.

[46] Fong SK, Kawash J, Wang Y, Johnson-Cicalese J, Polashock J, Vorsa N (2021). A low malic acid trait in cranberry fruit: genetics, molecular mapping, and interaction with a citric acid locus. Tree Genet Genomes.

[47] Panikashvili D, Savaldi-Goldstein S, Mandel T, Yifhar T, Franke RB, Höfer R (2007). The *Arabidopsis* DESPERADO/AtWBC11 transporter is required for cutin and wax secretion. Plant Physiol.

[48] Vogg G, Fischer S, Leide J, Emmanuel E, Jetter R, Levy AA (2004). Tomato fruit cuticular waxes and their effects on transpiration barrier properties: functional characterization of a mutant deficient in a very-long-chain fatty acid -ketoacyl-CoA synthase. J Exp Bot.

[49] Leide J, Hildebrandt U, Reussing K, Riederer M, Vogg G (2007). The developmental pattern of tomato fruit wax accumulation and its impact on cuticular transpiration barrier properties: effects of a deficiency in a β-ketoacyl-coenzyme A synthase (LeCER6). Plant Physiol.

[50] Moggia C, Graell J, Lara I, Schmeda-Hirschmann G, Thomas-Valdés S, Lobos GA (2016). Fruit characteristics and cuticle triterpenes as related to postharvest quality of highbush blueberries. Sci Hortic.

[51] Wang J, Hao H, Liu R, Ma Q, Xu J, Chen F (2014). Comparative analysis of surface wax in mature fruits between Satsuma mandarin (*Citrus unshiu*) and ‘Newhall’ navel orange (*Citrus sinensis*) from the perspective of crystal morphology, chemical composition and key gene expression. Food Chem.

[52] Yang Y, Zhou B, Zhang J, Wang C, Liu C, Liu Y (2017). Relationships between cuticular waxes and skin greasiness of apples during storage. Postharvest Biol Technol.

[53] Yang H, Zou Y, Li X, Zhang M, Zhu Z, Xu R (2022). QTL analysis reveals the effect of CER1-1 and CER1-3 to reduce fruit water loss by increasing cuticular wax alkanes in citrus fruit. Postharvest Biol Technol.

[54] Parsons EP, Popopvsky S, Lohrey GT, Lü S, Alkalai-Tuvia S, Perzelan Y (2012). Fruit cuticle lipid composition and fruit post-harvest water loss in an advanced backcross generation of pepper (*Capsicum* sp.). Physiol Plant.

[55] Park J-J, Jin P, Yoon J, Yang J-I, Jeong HJ, Ranathunge K (2010). Mutation in Wilted Dwarf and Lethal 1 (WDL1) causes abnormal cuticle formation and rapid water loss in rice. Plant Mol Biol.

[56] Jenks MA, Ashworth EN. Plant epicuticular waxes: Function, production, and genetics. In: Hortic Rev, vol 23. Oxford: Wiley; 2010. p. 1–54.

[57] Spayd SE, Tarara JM, Mee DL, Ferguson JC (2002). Separation of sunlight and temperature effects on the composition of *Vitis*
*vinifera* cv. Merlot berries Am J Enol Vitic.

[58] Ju Z, Bramlage WJ (2001). Developmental changes of cuticular constituents and their association with ethylene during fruit ripening in ‘Delicious’ apples. Postharvest Biol Technol.

[59] Belge B, Llovera M, Comabella E, Graell J, Lara I (2014). Fruit cuticle composition of a melting and a nonmelting peach cultivar. J Agric Food Chem.

[60] Belge B, Llovera M, Comabella E, Gatius F, Guillén P, Graell J (2014). Characterization of cuticle composition after cold storage of ‘Celeste’ and ‘Somerset’ sweet cherry fruit. J Agric Food Chem.

[61] Tafolla-Arellano JC, Zheng Y, Sun H, Jiao C, Ruiz-May E, Hernández-Oñate MA (2017). Transcriptome analysis of mango (*Mangifera*
*indica* L.) fruit epidermal peel to identify putative cuticle-associated genes. Sci Rep.

[62] Crisosto CH, Scott Johnson R, Luza JG, Crisosto GM (1994). Irrigation regimes affect fruit soluble solids concentration and rate of water loss of `O’Henry' peaches. HortScience.

[63] Baker EA, Procopiou J. The leaf and fruit cuticles of selected drought tolerant plants. In: International Symposium on Growth and Development of Fruit Crops, vol 27. 1997. p. 85–94.

[64] Tafolla-Arellano JC, Báez-Sañudo R, Tiznado-Hernández ME (2018). The cuticle as a key factor in the quality of horticultural crops. Sci Hortic.

[65] Wang W, Zhang Y, Xu C, Ren J, Liu X, Black K (2015). Cucumber ECERIFERUM1 (CsCER1), which influences the cuticle properties and drought tolerance of cucumber, plays a key role in VLC alkanes biosynthesis. Plant Mol Biol.

[66] Wang W, Liu X, Gai X, Ren J, Liu X, Cai Y (2015). *Cucumis*
*sativus* L. WAX2 plays a pivotal role in wax biosynthesis, influencing pollen fertility and plant biotic and abiotic stress responses. Plant Cell Physiol.

[67] Shepherd T, Griffiths DW (2006). The effects of stress on plant cuticular waxes. New Phytol.

[68] Rosenquist JK, Morrison JC (1989). Some factors affecting cuticle and wax accumulation on grape berries. Am J Enol Vitic.

[69] Solovchenko A, Merzlyak M (2003). Optical properties and contribution of cuticle to UV protection in plants: experiments with apple fruit. Photochem Photobiol Sci.

[70] Long LM, Patel HP, Cory WC, Stapleton AE (2003). The maize epicuticular wax layer provides UV protection. Funct Plant Biol.

[71] Al-Abdallat AM, Al-Debei HS, Ayad JY, Hasan S (2014). Over-expression of SlSHN1 gene improves drought tolerance by increasing cuticular wax accumulation in tomato. Int J Mol Sci.

[72] Schreiber L, Riederer M (1996). Ecophysiology of cuticular transpiration: comparative investigation of cuticular water permeability of plant species from different habitats. Oecologia.

[73] Sánchez FJ, Manzanares M, de Andrés EF, Tenorio JL, Ayerbe L (2001). Residual transpiration rate, epicuticular wax load and leaf colour of pea plants in drought conditions. Influence on harvest index and canopy temperature. Eur J Agron.

[74] Kin Pan Chung YZ, Jiang L (2016). COPII paralogs in plants: functional redundancy or diversity?. Trends Plant Sci.

[75] Fukuda N, Oshima Y, Ariga H, Kajino T, Koyama T, Yaguchi Y (2022). ECERIFERUM 10 encoding an enoyl-CoA reductase plays a crucial role in osmotolerance and cuticular wax loading in *Arabidopsis*. Front Plant Sci.

[76] Saharan GS, Mehta NK, Meena PD. Powdery Mildew disease of crucifers: biology, ecology and disease management. Springer Nature Singapore; 2019.

[77] Kuhn H, Kwaaitaal M, Kusch S, Acevedo-Garcia J, Wu H, Panstruga R (2016). Biotrophy at its best: novel findings and unsolved mysteries of the *Arabidopsis*-powdery mildew pathosystem. Arabidopsis Book.

[78] Uemura T, Kim H, Saito C, Ebine K, Ueda T, Schulze-Lefert P (2012). Qa-SNAREs localized to the trans-Golgi network regulate multiple transport pathways and extracellular disease resistance in plants. Proc Natl Acad Sci USA.

[79] McFarlane HE, Watanabe Y, Yang W, Huang Y, Ohlrogge J, Samuels AL (2014). Golgi- and trans-Golgi network-mediated vesicle trafficking is required for wax secretion from epidermal cells. Plant Physiol.

[80] Aarts MG, Keijzer CJ, Stiekema WJ, Pereira A (1995). Molecular characterization of the CER1 gene of *Arabidopsis* involved in epicuticular wax biosynthesis and pollen fertility. Plant Cell.

[81] Chen X, Goodwin SM, Boroff VL, Liu X, Jenks MA (2003). Cloning and characterization of the WAX2 gene of *Arabidopsis* involved in cuticle membrane and wax production. Plant Cell.

[82] Fiebig A, Mayfield JA, Miley NL, Chau S, Fischer RL, Preuss D (2000). Alterations in CER6, a gene identical to CUT1, differentially affect long-chain lipid content on the surface of pollen and stems. Plant Cell.

[83] Hansen JD, Pyee J, Xia Y, Wen TJ, Robertson DS, Kolattukudy PE (1997). The glossy1 locus of maize and an epidermis-specific cDNA from *Kleinia*
*odora* define a class of receptor-like proteins required for the normal accumulation of cuticular waxes. Plant Physiol.

[84] Xia Y, Nikolau BJ, Schnable PS (1996). Cloning and characterization of CER2, an *Arabidopsis* gene that affects cuticular wax accumulation. Plant Cell.

[85] Xia Y, Nikolau BJ, Schnable PS (1997). Developmental and hormonal regulation of the *Arabidopsis* CER2 gene that codes for a nuclear-localized protein required for the normal accumulation of cuticular waxes. Plant Physiol.

[86] Aharoni A, Dixit S, Jetter R, Thoenes E, Van Arkel G, Pereira A (2004). The SHINE clade of AP2 domain transcription factors activates wax biosynthesis, alters cuticle properties, and confers drought tolerance when overexpressed in *Arabidopsis*. Plant Cell.

[87] Moose SP, Sisco PH (1996). Glossy15, an APETALA2-like gene from maize that regulates leaf epidermal cell identity. Genes Dev.

[88] Tacke E, Korfhage C, Michel D, Maddaloni M, Motto M, Lanzini S (1995). Transposon tagging of the maize Glossy2 locus with the transposable element En/Spm. Plant J.

[89] Hannoufa A, Negruk V, Eisner G, Lemieux B (1996). The CER3 gene of *Arabidopsis*
*thaliana* is expressed in leaves, stems, roots, flowers and apical meristems. Plant J.

[90] Moose SP, Sisco PH (1994). Glossy15 controls the epidermal juvenile-to-adult phase transition in maize. Plant Cell.

[91] Evans MMS, Passas HJ, Scott Poethig R (1994). Heterochronic effects of glossy15 mutations on epidermal cell identity in maize. Development.

[92] Sturaro M, Hartings H, Schmelzer E, Velasco R, Salamini F, Motto M (2005). Cloning and characterization of GLOSSY1, a maize gene involved in cuticle membrane and wax production. Plant Physiol.

[93] Islam MA, Du H, Ning J, Ye H, Xiong L (2009). Characterization of Glossy1-homologous genes in rice involved in leaf wax accumulation and drought resistance. Plant Mol Biol.

[94] Bourdenx B, Bernard A, Domergue F, Pascal S, Léger A, Roby D (2011). Overexpression of *Arabidopsis* ECERIFERUM1 promotes wax very-long-chain alkane biosynthesis and influences plant response to biotic and abiotic stresses. Plant Physiol.

[95] Bernard A, Domergue F, Pascal S, Jetter R, Renne C, Faure J-D (2012). Reconstitution of plant alkane biosynthesis in yeast demonstrates that *Arabidopsis* ECERIFERUM1 and ECERIFERUM3 are core components of a very-long-chain alkane synthesis complex. Plant Cell.

[96] Lü S, Song T, Kosma DK, Parsons EP, Rowland O, Jenks MA (2009). *Arabidopsis* CER8 encodes LONG-CHAIN ACYL-COA SYNTHETASE 1 (LACS1) that has overlapping functions with LACS2 in plant wax and cutin synthesis. Plant J.

[97] Mao B, Cheng Z, Lei C, Xu F, Gao S, Ren Y (2012). Wax crystal-sparse leaf2, a rice homologue of WAX2/GL1, is involved in synthesis of leaf cuticular wax. Planta.

[98] Jetter R, Schäffer S, Riederer M (2000). Leaf cuticular waxes are arranged in chemically and mechanically distinct layers: evidence from *Prunus*
*laurocerasus* L. Plant Cell Environ.

[99] Martin LBB, Rose JKC (2014). There’s more than one way to skin a fruit: formation and functions of fruit cuticles. J Exp Bot.

[100] Croteau R, Fagerson IS (1971). The chemical composition of the cuticular wax of cranberry. Phytochemistry.

[101] Szakiel A, Pączkowski C, Pensec F, Bertsch C (2012). Fruit cuticular waxes as a source of biologically active triterpenoids. Phytochem Rev.

[102] Dzubak P, Hajduch M, Vydra D, Hustova A, Kvasnica M, Biedermann D (2006). Pharmacological activities of natural triterpenoids and their therapeutic implications. Nat Prod Rep.

[103] Shu C, Zhao H, Jiao W, Liu B, Cao J, Jiang W (2019). Antifungal efficacy of ursolic acid in control of *Alternaria*
*alternata* causing black spot rot on apple fruit and possible mechanisms involved. Sci Hortic.

[104] Jabeen K, Javaid A, Ahmad E, Athar M (2011). Antifungal compounds from *Melia*
*azedarach* leaves for management of *Ascochyta*
*rabiei*, the cause of chickpea blight. Nat Prod Res.

[105] Shaik AB, Ahil SB, Govardhanam R, Senthi M, Khan R, Sojitra R (2016). Antifungal effect and protective role of ursolic acid and three phenolic derivatives in the management of sorghum grain mold under field conditions. Chem Biodivers.

[106] Apel K, Hirt H (2004). Reactive oxygen species: metabolism, oxidative stress, and signaling. Annu Rev Plant Biol.

[107] Tadych M, Vorsa N, Wang Y, Bergen MS, Johnson-Cicalese J, Polashock JJ (2015). Interactions between cranberries and fungi: the proposed function of organic acids in virulence suppression of fruit rot fungi. Front Microbiol.

[108] Ding S, Zhang J, Yang L, Wang X, Fu F, Wang R (2020). Changes in cuticle components and morphology of ‘Satsuma’ mandarin (*Citrus*
*unshiu*) during ambient storage and their potential role on *Penicillium*
*digitatum* infection. Molecules.

[109] Tsuba M, Katagiri C, Takeuchi Y, Takada Y, Yamaoka N (2002). Chemical factors of the leaf surface involved in the morphogenesis of *Blumeria*
*graminis*. Physiol Mol Plant Pathol.

[110] Ringelmann A, Riedel M, Riederer M, Hildebrandt U (2009). Two sides of a leaf blade: *Blumeria*
*graminis* needs chemical cues in cuticular waxes of *Lolium*
*perenne* for germination and differentiation. Planta.

[111] Hansjakob A, Riederer M, Hildebrandt U (2011). Wax matters: absence of very-long-chain aldehydes from the leaf cuticular wax of the glossy11 mutant of maize compromises the prepenetration processes of *Blumeria*
*graminis*. Plant Pathol.

[112] Uppalapati SR, Ishiga Y, Doraiswamy V, Bedair M, Mittal S, Chen J (2012). Loss of abaxial leaf epicuticular wax in *Medicago*
*truncatula* irg1/palm1 mutants results in reduced spore differentiation of anthracnose and nonhost rust pathogens. Plant Cell.

[113] Schindelin J, Arganda-Carreras I, Frise E, Kaynig V, Longair M, Pietzsch T (2012). Fiji: an open-source platform for biological-image analysis. Nat Methods.

[114] Tattersall GJ. ThermImageJ: Thermal image functions and macros for ImageJ. 2019. 10.5281/zenodo.2652896.

[115] Catchen JM, Amores A, Hohenlohe P, Cresko W, Postlethwait JH (2011). Stacks: building and genotyping loci de novo from short-read sequences. G3.

[116] Li H, Durbin R (2009). Fast and accurate short read alignment with Burrows-Wheeler transform. Bioinformatics.

[117] Li H (2011). A statistical framework for SNP calling, mutation discovery, association mapping and population genetical parameter estimation from sequencing data. Bioinformatics.

[118] Broman KW, Wu H, Sen S, Churchill GA (2003). R/qtl: QTL mapping in experimental crosses. Bioinformatics.

[119] Robinson JT, Thorvaldsdóttir H, Wenger AM, Zehir A, Mesirov JP (2017). Variant review with the Integrative Genomics Viewer (IGV). Can Res.

[120] R Core Team. R: A language and environment for statistical computing. 2013. https://www.R-project.org/.

[121] Wickham H (2016). Ggplot2: elegant graphics for data analysis.

[122] Hebbali A. Olsrr: Tools for building OLS regression models. R package version 0.5.3. 2020. https://CRAN.R-project.org/package=olsrr.

[123] Mangiafico S. Rcompanion: Functions to support extension education program evaluation. R package version 2.4.13. 2022. https://CRAN.R-project.org/package=rcompanion.

[124] Mendiburu F, Yaseen M. Agricolae: Statistical procedures for agricultural research. R package version 1.4.0. 2020. https://CRAN.R-project.org/package=agricolae.

[125] Dunn OJ (1964). multiple comparisons using rank sums. Technometrics.

[126] Ogle DH, Doll JC, Wheeler P, Dinno A. FSA: Fisheries stock analysis. R package version 0.9.3. 2022. 10.5281/zenodo.6098468.

