# Supplementary material for: Cranberry fruit epicuticular wax benefits and identification of a wax-associated molecular marker
Source: BMC Plant Biol. 2023 Apr 5;23:181. doi: 10.1186/s12870-023-04207-w (PMC10074888; doi:10.1186/s12870-023-04207-w)
Supplement: Supplementary file 1 — Additional file 1: Figure S1. Examples of sound and sunscald cranberries in a field setting. [file 12870_2023_4207_MOESM1_ESM.pdf]

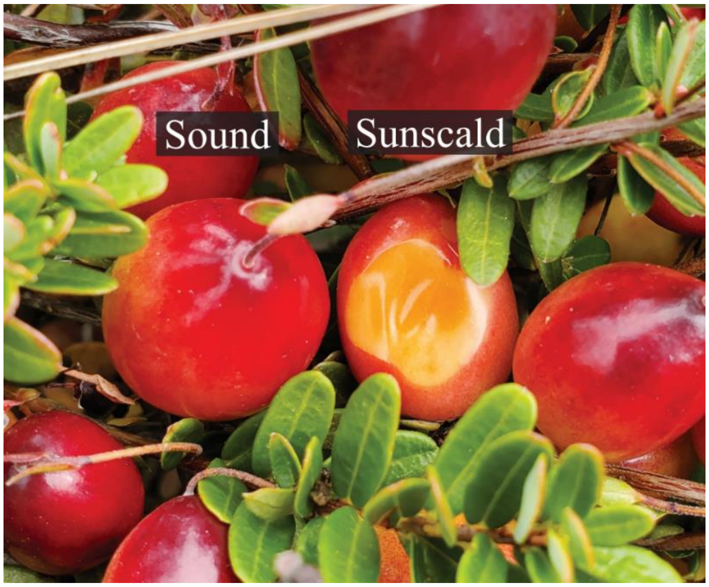

Figure S1. Examples of sound and sunscald cranberries in a field setting.

Sunscald in cranberries is attributed to an excess of heat, UV-radiation, and low humidity and is most likely to occur in the higher, sun-exposed regions of the cranberry canopy. An example of sunscald damage is shown here, where a temperature induced lesion has formed due to excess absorbed energy by pigmented cells. Secondary fungal infection and fruit rot often accompanies sunscald damage.
