# Supplementary material for: Cranberry fruit epicuticular wax benefits and identification of a wax-associated molecular marker
Source: BMC Plant Biol. 2023 Apr 5;23:181. doi: 10.1186/s12870-023-04207-w (PMC10074888; doi:10.1186/s12870-023-04207-w)
Supplement: Supplementary file 2 — Additional file 2: Figure S2. Pedigree for CNJ15-55, a cranberry population that segregates for epicuticular wax. Cranberry population CNJ15-55 was derived from a cross between CNJ08-103-20 and CNJ11-45-23 made in May 2015. Individuals within the CNJ15-55 population that set fruit were phenotyped for epicuticular wax and used in QTL analysis. Among these, a subset of 34 progeny with relatively high and low epicuticular wax was used for marker validation. [file 12870_2023_4207_MOESM2_ESM.pdf]

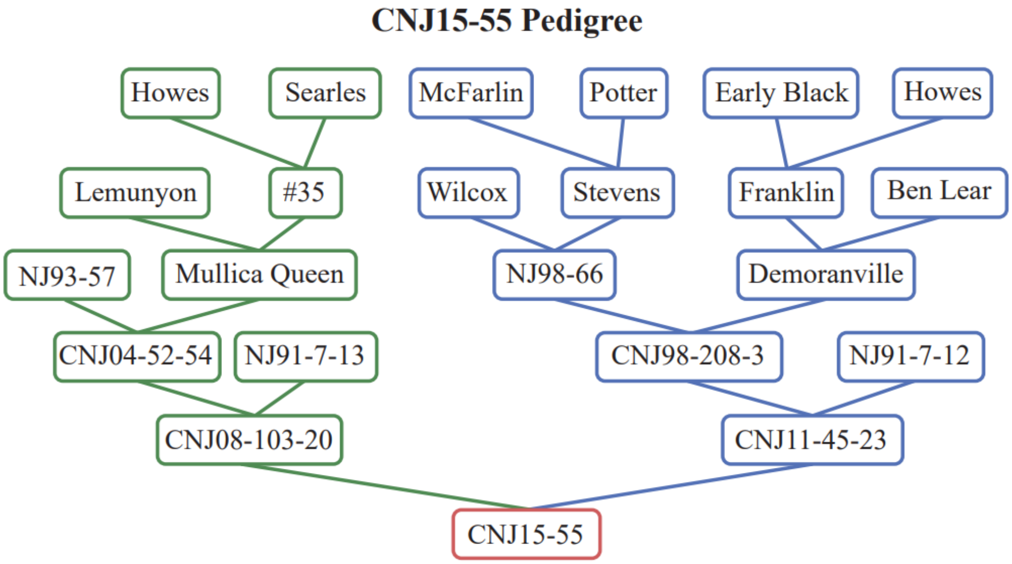

Figure S2: Pedigree for CNJ15-55, a cranberry population that segregates for epicuticular wax. Cranberry population CNJ15-55 was derived from a cross between CNJ08-103-20 and CNJ11-45-20 made in May 2015. Individuals within the CNJ15-55 population that set fruit were phenotyped for epicuticular wax and used in QTL analysis. Among these, a subset of 34 progeny with relatively high and low epicuticular wax was used for marker validation.
