# Supplementary figures and images for: Cranberry fruit epicuticular wax benefits and identification of a wax-associated molecular marker
Source: BMC Plant Biol. 2023 Apr 5;23:181. doi: 10.1186/s12870-023-04207-w (PMC10074888; doi:10.1186/s12870-023-04207-w)

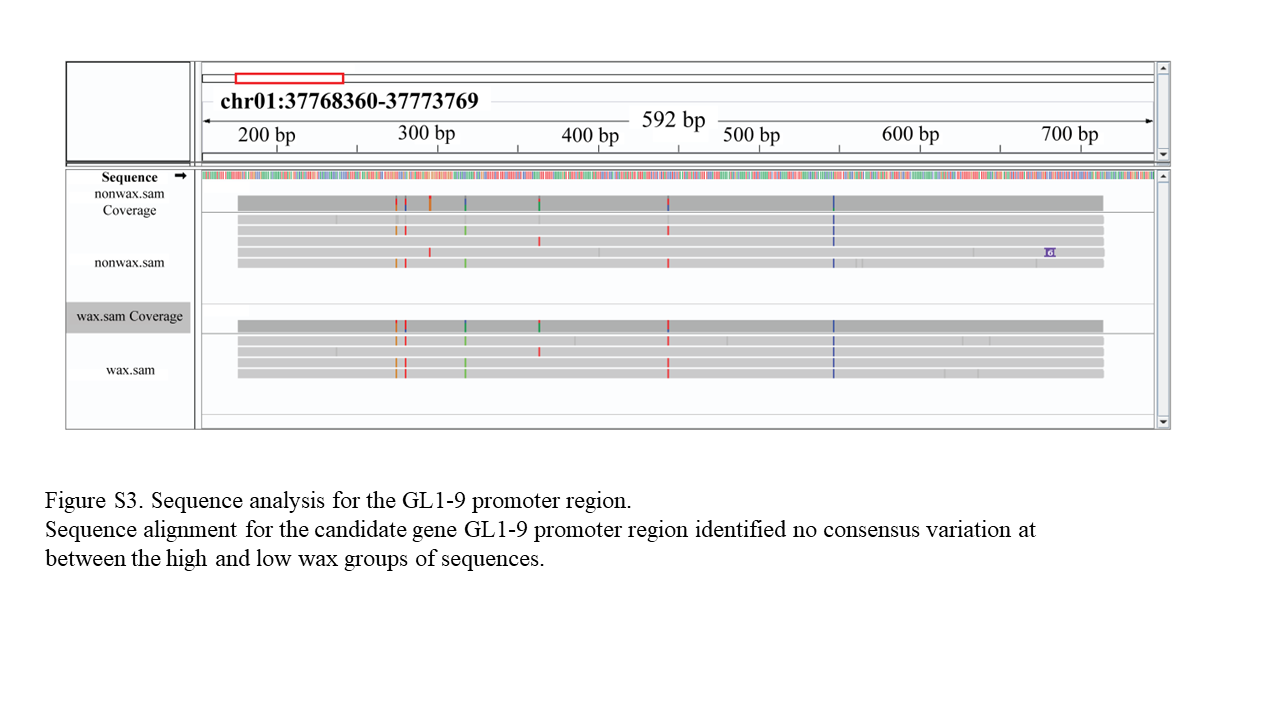

Supplement: Supplementary file 3 — Additional file 3. [file 12870_2023_4207_MOESM3_ESM.png]
